# Bacterial Responses to Glyoxal and Methylglyoxal: Reactive Electrophilic Species

**DOI:** 10.3390/ijms18010169

**Published:** 2017-01-17

**Authors:** Changhan Lee, Chankyu Park

**Affiliations:** Department of Biological Sciences, Korea Advanced Institute of Science and Technology, Yuseong-gu, Daejeon 305-701, Korea; nasa@kaist.ac.kr

**Keywords:** glyoxal, reactive electrophilic species (RES), glyoxalase

## Abstract

Glyoxal (GO) and methylglyoxal (MG), belonging to α-oxoaldehydes, are produced by organisms from bacteria to humans by glucose oxidation, lipid peroxidation, and DNA oxidation. Since glyoxals contain two adjacent reactive carbonyl groups, they are referred to as reactive electrophilic species (RES), and are damaging to proteins and nucleotides. Therefore, glyoxals cause various diseases in humans, such as diabetes and neurodegenerative diseases, from which all living organisms need to be protected. Although the glyoxalase system has been known for some time, details on how glyoxals are sensed and detoxified in the cell have not been fully elucidated, and are only beginning to be uncovered. In this review, we will summarize the current knowledge on bacterial responses to glyoxal, and specifically focus on the glyoxal-associated regulators YqhC and NemR, as well as their detoxification mediated by glutathione (GSH)-dependent/independent glyoxalases and NAD(P)H-dependent reductases. Furthermore, we will address questions and future directions.

## 1. Introduction

**Figure 1:**
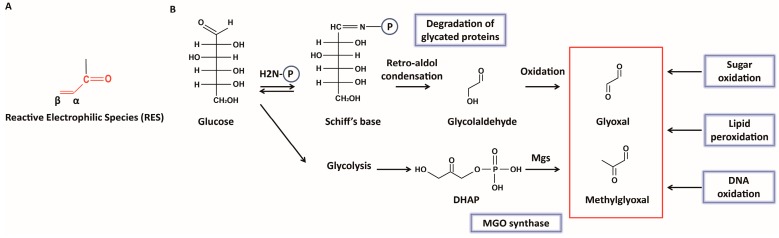
Chemical structures of reactive electrophilic species (RES) and the formation of glyoxals. (**A**) RES are compounds containing α,β-unsaturated carbonyl (colored in red) or other electrophilic groups; (**B**) Glyoxal (GO) and methylglyoxal (MGO) are produced in cells. Glucose forms a Schiff’s base with the amino group of protein (H2N-P), and subsequent retro-aldol condensation produces glycolaldehyde [8]. Oxidation of glycolaldehyde generates GO [8].Various sugars (including glucose) generate dihydroxyacetone phosphate (DHAP), which is converted to MGO by MGO synthase (Mgs) [9]. In addition, sugar oxidation, lipid peroxidation, and DNA oxidation are involved in the production of glyoxals [10,11].

Reactive electrophilic species (RES) are compounds containing α,β-unsaturated carbonyl or other electrophilic groups (Figure 1A) [1]. Because of their reactivity, they interact with macromolecules and affect cellular redox, mediated by redox cofactors like glutathione (GSH) and NAD(P)H [2,3]. The results often involve cellular malfunction. α-Oxoaldehydes—glyoxal (GO) and methylglyoxal (MGO), containing two reactive carbonyl groups (Figure 1B)—are examples of RES. Although stress responses to reactive oxygen species (ROS) and nitrogen species (RNS) have been extensively studied, research on the response to RES have emerged only recently. GO and MGO were reported to be associated with aging and diseases such as diabetes, Alzheimer’s, and Parkinson’s diseases [4,5,6]. However, cellular responses to glyoxals are largely unknown. Although the major glyoxal-detoxification systems using glutathione are present in most species from bacteria to humans [7], a number of issues on sensing and detoxification of glyoxals still remains to be investigated. Recently, considerable efforts have been made to study cellular responses to glyoxals for bacteria as a model system in terms of their formation, cytotoxic targets, and detoxification. In this review, we will summarize recent findings on cellular responses to GO/MGO and its detoxification.

## 2. Formation of Glyoxals

GO and MGO are primarily produced from the oxidative degradation of glucose [8], and lipid peroxidation and DNA oxidation are also known as a source of glyoxals (Figure 1B) [10,11]. Glycation is a reversible modification of proteins capable of generating glyoxals [8]. MGO is produced from a glycolysis intermediate—dihydroxyacetone phosphate (DHAP)—which is converted to MGO by MGO synthase (Mgs) (Figure 1B) [9,12]. MGO is also generated during l-threonine degradation [13].

## 3. Bacterial Sensing of Glyoxals

Although detoxification pathways have been relatively well studied, the initial responsive element for glyoxals has rarely been characterized. Recently, several transcriptional regulators responding to glyoxal/electrophiles have been investigated [14,15]. Here, we describe recent findings regarding glyoxal detection in bacteria.

### 3.1. YqhC, a Major Regulator for Glyoxal (GO)-Detoxifying YqhDE

YqhC belongs to the AraC/XylS transcriptional regulators, acting as an activator. *YqhC* is an outwardly transcribing gene from *yqhDE*, with presumably overlapping promoter regions between them. *YqhC* was discovered as a gene for GO-resistant allele [14], in which a number of GO-resistant mutations were isolated by growing bacterial cells on an Luria-Bertani (LB) medium plate containing a lethal concentration of GO [14]. The mutation overexpresses YqhD aldehyde reductase, which is a major GO-detoxifying enzyme [14]. YqhC directly binds to the promoter region of *yqhD* [14], which is activated by GO and furfural [14,16]. *YqhE*—encoding aldo-keto reductase—is also upregulated by furfural via YqhC [16]. However, it is still unclear as to whether these electrophiles affect YqhC directly. *YqhC* and *yqhDE* genes are found not only in species closely related to *Escherichia coli*, but also in more distant Gram-negative bacteria [16], implying that the *yqhC–yqhDE* detoxification system may provide a universal mechanism for Gram-negative bacteria for the detoxification of electrophiles, including GO and MGO.

### 3.2. NemR, a Potential Reactive Electrophilic Species (RES) Sensor for Regulating the nemRA-gloA Operon

NemR belongs to TetR family of transcriptional factors [17]. The *nemR* gene is a member of an operon containing *nemA*, encoding a *N*-ethylmaleimide reductase in its downstream [17]. *N*-ethylmaleimide is a thiol scavenger with strong electrophilic nature. The transcriptional coordination between *nemRA* and *gloA* genes was recently reported [15,18]. A mutation in the NemR-binding site of the *nemRA* operon was also isolated with a GO-resistant phenotype [15]. Since the *gloA* gene is a member of the *nemRA* operon, a point mutation at the NemR-binding site results in over-expression of GloA, thereby conferring resistance to glyoxal. Consistently, a transcriptomic analysis revealed that the *nemRA* and *gloA* genes are co-expressed upon treatment with MGO [15,18]. NemA is a member of the old yellow enzymes with flavin mononucleotide (FMN) as a cofactor, reducing various aldehyde compounds, such as glyoxals and quinones [19,20]. Quinones are electrophilic, serving as electron donors in the respiratory system. DNA binding affinity of NemR decreases upon the addition of GO/MGO or quinones, but not with their reduction products (i.e., 1,2-ethandiol/1,2-propanediol and quinol) [15]. In addition, NemR serves as a sensor for reactive chlorine species (RCS), which are strong oxidants with electrophilic activity [21,22]. NemR appears to be a specialized sensor for RES, because the DNA-binding affinity and transcription level of the *nemRA-gloA* operon is not affected by ROS [15]. On the other hand, the transcriptional regulator RutR binds to the upstream region of the *nemRA-gloA* operon, although it does not play a role in the regulation of *nemRA-gloA* [15,17].

Cysteine residues play a crucial role in *nemRA-gloA* regulation. Among six cysteine residues (21, 98, 106, 116, 149, and 153) of *E. coli* NemR, two at 21 and 116 were demonstrated to be essential in responding to electrophiles (including GO and MGO), in which formation of intermolecular disulfide bonds involving these residues were required both in vivo and in vitro [15]. RES and glyoxals oxidize cysteines, thereby forming disulfide bonds. This type of redox regulation is basically reversible, such that an oxidized NemR can be reactivated by a reducing agent. However, the cysteine at position 106 that is conserved among NemR homologs has been identified as a critical residue for responding to RCS, such as hypochlorous acid [21]. Recently, the formation of a Cys106-Lys175 sulfenamide bond was shown to serve as a thiol-based redox switch to modulate DNA-binding of NemR in response to RCS [23]. It is of interest to note that different stress conditions involve modifications of different cysteine residues to regulate the activity of NemR. In summary, NemR functions as an RES-specific regulator through various modifications (such as alkylation and oxidation) of cysteine residues.

### 3.3. CRP/cAMP-Mediated Detoxification Pathway for Glyoxal

CRP is a global transcriptional regulator for more than 180 genes, and requires cAMP [24]. The *crp* mutant was recently found to be associated with GO resistance [25], suggesting that cAMP and CRP are involved in responding to GO and its detoxification. Consistently, a mutation of *cya* gene encoding adenylate cyclase has been shown to confer resistance to GO/MGO [25]. From the study of genes regulated by cAMP/CRP (which are directly involved in the detoxification of GO/MGO), we were able to reveal new members of cAMP/CRP regulon: *yqhC* (transcriptional regulator), *yqhD* (NADPH-dependent aldehyde reductase), *yafB* (NADPH-dependent aldo-keto reductase), *sodB* (superoxide dismutase), and *gloA* (glyoxalase I). These genes are negatively regulated by CRP. In addition to cAMP/CRP, the *gloA* gene is also known to be regulated by NemR when it is expressed as part of the *nemRA-gloA* operon. It was shown that the level of *nemRA* transcript was unaffected by CRP. Therefore, it was inferred that CRP only affects *gloA* by acting on its own promoter [15,25]. Conclusively, it was found that *gloA* is regulated by dual promoters with two different transcriptional regulators. Since the cAMP level and glucose uptake are inversely correlated through the phosphotransferase system (PTS) [26], an expression of glyoxalase I might be necessary for the reduction of intracellular glyoxals produced from the oxidative degradation of glucose.

### 3.4. Fnr and NsrR—Regulators of YafB Aldo-Keto Reductase

Aldo-keto reductases (AKRs) play a crucial role in the detoxification of glyoxals by reducing GO and MGO to glycolaldehyde and acetol, respectively [27,28]. Among the several AKRs of *E. coli*, the YafB protein is a major one, based on the fact that the screening of GO-resistant mutant from the strain lacking *yqhD* and *gloA* yielded a number of mutants overexpressing YafB through genomic rearrangements, such as multi-base deletions and recombination in the upstream region of the *yafB* gene [29]. As a result of the genomic rearrangements, transcriptional fusions of *yafB* to the upstream *rrn* occurred. In addition, the negative regulatory sites for NsrR and Fnr were removed, resulting in enhancement of YafB expression [29]. NsrR is a nitrite-sensitive transcription repressor sensing nitrite due to its iron-sulfur [2Fe-2S] cluster essential for sensing redox change [30]. Fnr is a redox-responsive transcriptional factor harboring [4Fe-4S] cluster to sense oxygen and nitric oxide [31]. Fnr is also involved in the activation and repression of genes in anaerobic and aerobic metabolism [31,32]. YafB expression is increased upon GO treatment by de-repressing NsrR/Fnr [29]. However, the de-repression by NsrR/Fnr is directly influenced by GO or indirectly by redox imbalance caused by GO. Interestingly, among AKRs, transcripts of *yghZ* and *yajO* are reduced upon exposure to GO [28]. This may indicate the presence of regulatory divergence in various stress conditions.

## 4. Detoxification of Glyoxals

**Figure 2:**
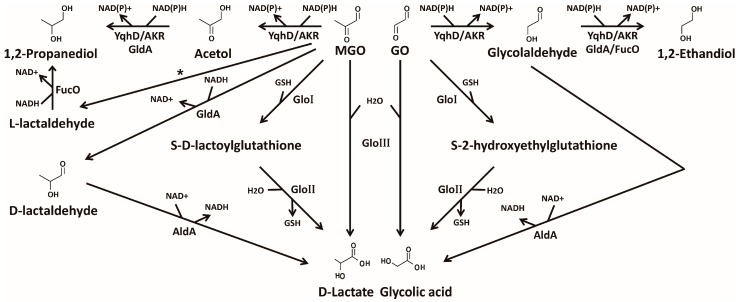
Detoxification of glyoxals. Detoxification of GO/MGO are primarily mediated by glyoxalases and NAD(P)H-dependent enzymes. Glyoxalase I and II (GloI and II) utilize glutathione (GSH) to convert glyoxals to acid species. Glyoxalase III (GloIII) catalyzes the same reaction, but without any cofactors [33,34]. Various aldo-keto reductases (AKRs) and YqhD are involved in the reduction of glyoxals to alcoholic species with NAD(P)H [14,27,28]. FucO and GldA are also involved in glyoxal metabolism by reducing l-lactaldehyde/glycoladehyde and acetol/glycolaldehyde, respectively [12,35]. GldA converts MGO to d-lactaldehyde [12], while glycolaldehyde and d-lactaldehyde are oxidized to glycolic acid and d-lactate, respectively, by AldA (aldehyde dehydrogenase) [36]. The suspected activity is marked with an asterisk [37].

Living organisms employ various means of detoxifying reactive GO and MGO. Bacterial detoxifications are carried out primarily by the following two systems: glyoxalase and NAD(P)H-dependent detoxification enzyme (Figure 2). Glyoxalases are further classified based on their cofactor requirement; GSH-dependent or -independent. Except for the well-known glyoxalase I/II, other enzymes associated with GO/MGO are listed in Table 1 with their enzymatic characteristics.

### 4.1. GSH-Dependent Glyoxalase System

Glutathione—a redox cofactor—contains cysteine, with its thiol group that is crucial for activity. Bacterial cells utilize GSH for the detoxification of glyoxals, which is mediated by two proteins: GloA (glyoxalase I) and GloB (glyoxalase II) [7]. GO and MGO can be converted by GloA to *S*-2-hydroxyethylglutathione and *S*-d-lactoylglutathione, respectively, which are the intermediates of the GSH-dependent pathway [7]. Notably, these intermediates modulate the activity of the potassium efflux pump [38,39]. An acidification of the cytoplasm through the KefB and KefC systems is known to protect bacterial cells from glyoxal toxicity [40]. The precise mechanism of protection by cytoplasmic acidification is poorly understood, although it was suggested that this change is to minimize glyoxal-induced DNA damage [39]. GloB converts the above intermediates to glycolic and lactic acids. Therefore, an availability of GSH would be critical in the proper functioning of the GloAB system in the cell. *Bacillus subtilis*—a Gram-positive bacterium—has a similar glyoxalase system, but utilizes bacillithiol (BSH) instead of GSH [41]. While GSH is synthesized from l-cysteine, l-glutamic acid, and glycine, BSH consists of l-cysteinyl-d-glucosamine and malic acid.

### 4.2. GSH-Independent Glyoxalase System

**Figure 3:**
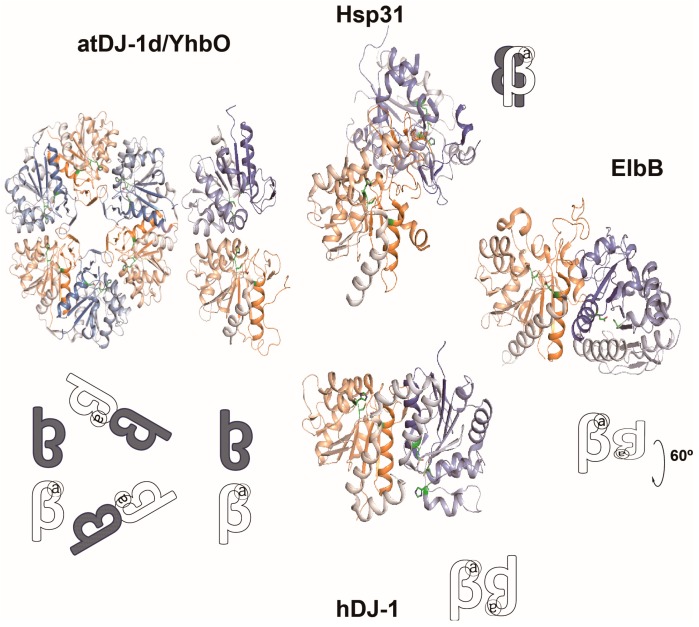
Structural and oligomeric diversities of DJ-1 homologs. Dimeric or hexameric structure of atDJ-1d (PDB code: 4OFW)/YhbO (PDB code: 1OI4), Hsp31 (PDB code: 1N57), ElbB (PDB code: 1OY1), and hDJ1 (PDB code: 1UCF, similar to *E. coli* YajL) are shown. DJ-1 homologs share α/β core domain. Formations of oligomeric structures are schematized with the letter “β” as a monomer and “a” as the catalytic site.

From the previous report on the activity of GSH-independent glyoxalase [42], the gene encoding the activity of glyoxalase III was identified [33,43]. This enzyme catalyzes the same reaction as that of glyoxalase I/II enzymes without any cofactor. An *E. coli* homolog of glyoxalase III was first discovered by the activity tracing of fractionated *E. coli* extract, leading to a characterization of the *hchA* (Hsp31) gene for GSH-independent glyoxalase III [33]. The Hsp31 protein is a member of the DJ-1 homologs that are present in all biological species [33]. In *E. coli*, there are four homologs: *hchA*, *yajL*, *yhbO*, and *elbB*. The Cys-185 and Glu-77 residues of Hsp31 and their corresponding positions in other homologs are essential to catalysis [34]. Hsp31 and YhbO harbor an additional His residue at 186 [34]. YajL and ElbB have Phe and Ile residues, respectively, instead of His [34]. The DJ-1 homologs share structural similarity of central β-strands surrounded by α-helices, forming a dimer or an oligomer (Figure 3) [34,44]. Analogous to atDJ-1d of *Arabidopsis thaliana*, YhbO likely forms a circular structure of a hexamer being composed of three dimers [44,45,46]. Purified Hsp31, YajL, YhbO, and ElbB show glyoxalase activity with different substrate specificity (Table 1) [33,34]. Over-expressions of the homologs exhibit protection against exogenously added GO/MGO, and reduce the glyoxal-dependent accumulation of advanced glycation end products (AGEs). Glycation is a nonenzymatic reaction between protein and sugar/glyoxal, presumably resulting in protein malfunction. Recent reports propose that Hsp31 and human DJ-1 protein are deglycases likely associated with protein repair [47,48].

### 4.3. NAD(P)H-Dependent Detoxifying Enzymes

The electron donor NAD(P)H is a redox cofactor for various enzymes, including ones for glyoxal detoxification. YqhD is an NADPH-dependent aldehyde reductase converting GO/MGO to corresponding alcohols (Figure 2, Table 1) [14,49]. As described above, the existence of YqhD enzyme was revealed by characterizing GO-resistant mutations with constitutively-activating YqhC transcriptional regulator, resulting in YqhD overexpression [14]. YqhD turned out to be a major detoxification pathway for GO [14]. Although YqhD exhibits activity toward MGO, it does not play a major role in MGO detoxification. Instead, glyoxalase I contributes more in vivo [14,15]. AKRs are also NADPH-dependent enzymes, converting GO/MGO to less-reactive alcoholic species (Figure 2, Table 1) [27,28]. The *E. coli* genome encodes nine AKRs, among which five of them—YqhE, YafB, YghZ, YeaE, and YajO—show activity to GO/MGO [28]. The role of AKRs in glyoxal metabolism is likely to be multi-faceted in response to various physiological changes; i.e., intracellular concentrations of glyoxals, other metabolites, or growth states. Since the AKRs have different kinetic and expression profiles in relation to the intracellular concentration of glyoxal, they might have unique roles in the detoxification of glyoxal. YqhD and AKRs exhibit two-step reductions of glyoxals, converting GO/MGO to 1,2-ethandiol/1,2-propanediol via glycolaldehyde/acetol, respectively [14,27,28]. The first step is critical in decreasing the toxicity of glyoxal. FucO (1,2-propanediol oxidoreductase) is involved in the conversion of l-lactaldehyde and glycolaldehyde to 1,2-propanediol and 1,2-ethandiol, respectively [35,50]. GldA (glycerol dehydrogenase) is involved in NADH-dependent reductions of glycolaldehyde and acetol [12]. GldA also converts MGO to d-lactaldehyde, and AldA (aldehyde dehydrogenase) oxidizes glycolaldehyde and d-lactaldehyde to glycolic and d-lactic acids, respectively, using NAD^+^ as a cofactor [12,35,36] (Figure 2).

## 5. Toxicity of Glyoxals

Since GO and MGO are highly reactive (especially to nucleophilic macromolecules, including proteins and nucleotides), strong intracellular toxicity has been found. To protect from glyoxal toxicity, cellular redox cofactors such as GSH and NAD(P)H are required, without which cells are endangered with irreversible damages.

### 5.1. Protein Damage

Since the carbonyl groups of glyoxal are highly reactive, they interact with diverse molecules including proteins and nucleotides. GO and MGO form a Schiff’s base with the amino group of amino acids, and subsequent Amadori rearrangement produces AGEs [5]. The nucleophilic residues of proteins, such as lysine, cysteine, arginine, and histidine, are prone to react with GO/MGO. Carboxymethyl- and carboxyethyl-lysines (CML/CEL) or products with other amino acids are formed from reactions with GO and MGO, respectively [51,52]. The spontaneous non-enzymatic glycation affects protein function. The glycation itself is basically reversible, although the reversal of AGE formation is still controversial.

### 5.2. Nucleotide Damage

Nucleotides are vulnerable targets of GO/MGO, forming adducts such as carboxymethyl- and carboxyethyl guanosine (CMG/CEG) to increase mutation frequency [53,54]. However, the mechanism underlying nucleotide modification caused by glyoxals is largely unknown. Recent study indicates that genes associated with DNA repair (*recA*, *recC*) and tRNA modification (*truA*, *trmE*, *gidA*) are involved in GO sensitivity, suggesting that nucleotides are one of the main targets of GO [25]. The repair of DNA damage and tRNA modification seem to be crucial in enduring GO assault. RecA catalyzes an exchange of DNA strands in the recombinational repair, and also cleaves LexA repressor for an induction of the SOS response [55]. As a subunit of RecBCD exonuclease V, RecC is necessary for homologous recombination in repairing double strand break [55]. Therefore, the isolation of *recA* and *recC* mutations as GO-sensitive derivatives suggests that the strand break of DNA might occurs with GO, which is likely to be repaired by the recombinational repair system. The *trmE*, *truA*, and *gidA* genes are involved in tRNA stability [56]. Thus, it is likely that unmodified tRNA is more sensitive to GO than modified tRNA. Alternatively, since the tRNA modification stabilizes the U·G pairing at the wobble position and plays a role in decoding NNG codons [57], wobble pairing in translation may serve as a target for GO. The mutant strains related to DNA repair show comparable sensitivity to both GO and MGO, but mutations in tRNA modification exert more sensitivity to GO than to MGO [25]. Therefore, tRNA is more likely to interact with GO than MGO, implying different target specificity of glyoxal species.

### 5.3. Relation to Oxidative Stress

ROS have been known for some time and are reasonably well-characterized. Since the detoxification of RES (including glyoxals) requires several redox cofactors (such as NAD(P)H and GSH), it may affect redox regulation associated with electron transport chain, which is a site for ROS production. In eukaryotes, a decrease in the activity of the respiratory complex III caused by MGO glycation results in oxidative stress [58]. A removal of aldehyde is closely associated with oxidative stress—e.g., glycolaldehyde-dependent induction of SoxRS regulon [59]. Recent study of genetic screening revealed SodB (superoxide dismutase) as one of the GO-resistance genes under the regulation of CRP [25]. On the other hand, a high level of oxidative stress increases the production of glyoxals, presumably via an oxidative degradation of sugar [8]. The other possibility would be an oxidation of aldehyde, which also likely generates glyoxal, especially under the condition lacking superoxide dismutase [60]. Finally, the expression of glyoxalase exhibits protection from glyoxal as well as oxidative stress [61,62]. Therefore, ROS and RES are somehow closely associated in vivo.

## 6. Conclusions and Perspectives

**Figure 4:**
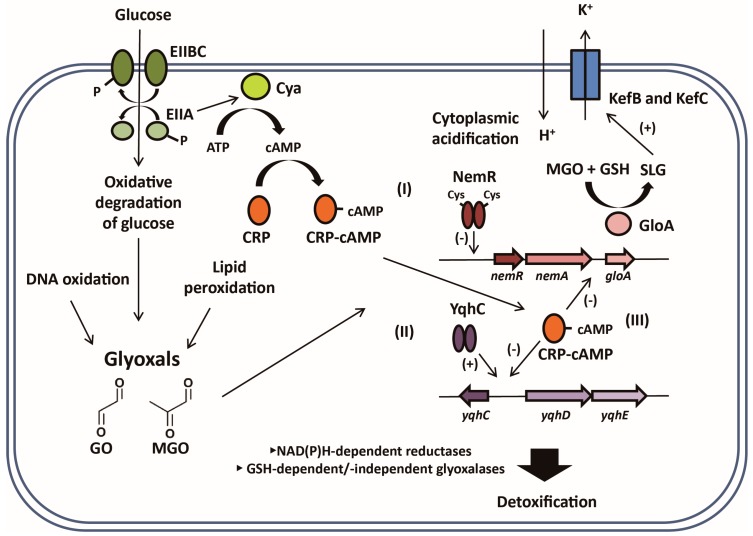
Cellular response to glyoxals. GO and MGO accumulated from various sources are detected by target agents. (**I**) NemR recognizes RES and glyoxals via the oxidation of cysteine residues, and the *nemRA-gloA* operon is subsequently de-repressed; (**II**) YqhC upregulates YqhD aldehyde reductase and YqhE aldo-keto reductase in response to glyoxal; (**III**) cAMP/CRP negatively regulates the expression of its regulons involved in GO detoxification (i.e., *yqhCD* and *gloA*). Enzyme IIB and C (EIIBC) in the phosphotransferase system (PTS) dephosphorylate Enzyme IIA (EIIA) upon glucose uptake. When the glucose concentration is high, EIIA is dephosphorylated, thereby reducing the activity of adenylate cyclase (Cya). For the detoxification of GO/MGO, NAD(P)H-dependent enzymes and glyoxalases—either GSH-dependent or -independent—are involved. In addition, acidification via KefB and KefC potassium efflux systems contributes to cell survival against glyoxal toxicity. KefB and KefC are activated by *S*-d-lactoylglutathione (SLG) produced by GloA (glyoxalase I).

The production of GO/MGO in vivo appears to be inevitable, since they are intrinsically associated with various physiological processes. Like ROS, RES are fairly reactive, thereby resulting in stress to the cell. Due to their toxic effect, the cell has devised various enzyme systems to reduce their toxicity. Our current knowledge only focuses on individual detoxification pathways, without an organized scheme to systematically detect their actions and respond appropriately. Ironically, bacteria have an MGO producing enzyme—MGO synthase [9]. This review attempted to delineate global processes responding to glyoxal/RES (Figure 4). A systematic characterization of responsive and regulatory players for glyoxals was only recently conducted in bacteria. It is expected that much of the future novelty lies in this aspect of RES response. Meanwhile, since the toxicity of RES is closely associated with various human diseases (particularly neurodegenerative diseases), a fundamental understanding of RES response obtained from the study of prokaryotes can be applied to higher eukaryotes for the development of novel therapy. Although we learned that ROS and RES are intimately associated, the detailed mechanisms of how they relate to each other are yet to be investigated. The molecular mechanisms involving the cAMP- and YqhC/NemR-mediated RES sensors also need to be unraveled, in addition to the effect of glyoxal on DNA damage and tRNA modification. Finally, the ubiquitous presence of DJ-1 homologs (we propose the name “glyoxalin”, as in “peroxiredoxin”) and numerous AKRs present an issue of redundancy in single organism. Addressing these questions with regard to their specified roles and expression patterns would be one of the future challenges.

## Figures and Tables

**Table 1:** Enzymatic constants for GO/MGO detoxification proteins.

Proteins	Substrate	Product	*K*_m_ (mM)	*k*_cat_ (min^−1^)	*k*_cat_/*K*_m_ (min^−1^·M^−1^)	Reference
YqhD	GO	Glycolaldehyde	11.53	618	5.36 × 10^4^	[14]
	Glycolaldehyde	1,2-Ethandiol	28.28	3258	1.09 × 10^5^	[14]
	MGO	Acetol	2.6	284	1.09 × 10^5^	[14]
	Acetol	1,2-Propanediol	76.9	144	1.87 × 10^3^	[14]
YqhE	GO	Glycolaldehyde	22	296	1.34 × 10^4^	[28]
	Glycolaldehyde	1,2-Ethandiol	12	15	1.25 × 10^3^	[28]
	MGO	Acetol	2.05	1657	8.09 × 10^9^	[27]
YafB	GO	Glycolaldehyde	60	368	6.13 × 10^3^	[28]
	Glycolaldehyde	1,2-Ethandiol	17	22	1.29 × 10^3^	[28]
	MGO	Acetol	2.46	1749	7.13 × 10^5^	[27]
YghZ	GO	Glycolaldehyde	104	458	4.40 × 10^3^	[28]
	Glycolaldehyde	1,2-Ethandiol	104	140	1.34 × 10^3^	[28]
	MGO	Acetol	6.91	661	0.96 × 10^5^	[27]
YeaE	GO	Glycolaldehyde	10	15	1.50 × 10^3^	[28]
	Glycolaldehyde	1,2-Ethandiol	7	15	2.14 × 10^3^	[28]
	MGO	Acetol	2.09	171	0.82 × 10^5^	[27]
YajO	GO	Glycolaldehyde	N.D.	N.D.	N.D.	[28]
	Glycolaldehyde	1,2-Ethandiol	N.D.	N.D.	N.D.	[28]
	MGO	Acetol	N.D.	N.D.	N.D.	[27]
NemA	GO	Glycolaldehyde	318.88	1008	3.15 × 10^3^	[15]
	MGO	Acetol	25.63	1276	4.98 × 10^4^	[15]
Hsp31	GO	Glycolaldehyde	5.94	16	2.78 × 10^3^	[33]
	MGO	Acetol	1.43	156	1.09 × 10^5^	[33]
YhbO	GO	Glycolaldehyde	2.97	70	3.11 × 10^5^	[34]
	MGO	Acetol	0.06	20.8	3.47 × 10^5^	[34]
YajL	GO	Glycolaldehyde	2.97	70	0.24 × 10^5^	[34]
	MGO	Acetol	N.D.	N.D.	N.D.	[34]
ElbB	GO	Glycolaldehyde	1.21	0.48	0.40 × 10^3^	[34]
	MGO	Acetol	N.D.	N.D.	N.D.	[34]
GldA	MGO	d-lactaldehyde	0.5	0.1	2.03 × 10^2^	[12]
	Glycolaldehyde	1,2-Ethandiol	0.85	0.53	6.23 × 10^2^	[12]
	Acetol ^a^	1,2-Propanediol	N.D.	N.D.	N.D.	[12]

N.D. (Not detected); ^a^ GldA (glycerol dehydrogenase) shows specific activity to acetol as 4600 nmol·min^−1^·mg^−1^ [12].
